# Pooled resequencing of larvae and adults reveals genomic variations associated with Ostreid herpesvirus 1 resistance in the Pacific oyster *Crassostrea gigas*

**DOI:** 10.3389/fimmu.2022.928628

**Published:** 2022-08-19

**Authors:** Shanshan Yao, Li Li, Xudong Guan, Yan He, Aude Jouaux, Fei Xu, Ximing Guo, Guofan Zhang, Linlin Zhang

**Affiliations:** ^1^ Chinese Academy of Sciences (CAS) and Shandong Province Key Laboratory of Experimental Marine Biology and Center of Deep Sea Research, Center for Ocean Mega-Science, Institute of Oceanology, Chinese Academy of Sciences, Qingdao, China; ^2^ Laboratory for Marine Biology and Biotechnology, Qingdao National Laboratory for Marine Science and Technology, Qingdao, China; ^3^ College of Life Sciences, Qingdao University, Qingdao, China; ^4^ University of Chinese Academy of Sciences, College of Marine Science, Beijing, China; ^5^ Ministry of Education (MOE) Key Laboratory of Molecular Genetics and Breeding, College of Marine Life Sciences, Ocean University of China, Qingdao, China; ^6^ UMR BOREA, “Biologie des Organismes et Ecosystèmes Aquatiques”, MNHN, UPMC, UCBN, CNRS-7208, IRD, Université de Caen Basse-Normandie, Esplanade de la Paix, Caen, France; ^7^ Haskin Shellfish Research Laboratory, Department of Marine and Coastal Sciences, Rutgers University, Port Norris, NJ, United States

**Keywords:** Ostreid herpesvirus, antiviral innate immunity, oyster, pooled-resequencing, transcriptomic response, disease resistance, larval mortality, molluscan aquaculture

## Abstract

The Ostreid herpesvirus 1 (OsHV-1) is a lethal pathogen of the Pacific oyster (*Crassostrea gigas*), an important aquaculture species. To understand the genetic architecture of the defense against the pathogen, we studied genomic variations associated with herpesvirus-caused mortalities by pooled whole-genome resequencing of before and after-mortality larval samples as well as dead and surviving adults from a viral challenge. Analysis of the resequencing data identified 5,271 SNPs and 1,883 genomic regions covering 3,111 genes in larvae, and 18,692 SNPs and 28,314 regions covering 4,863 genes in adults that were significantly associated with herpesvirus-caused mortalities. Only 1,653 of the implicated genes were shared by larvae and adults, suggesting that the antiviral response or resistance in larvae and adults involves different sets of genes or differentiated members of expanded gene families. Combined analyses with previous transcriptomic data from challenge experiments revealed that transcription of many mortality-associated genes was also significantly upregulated by herpesvirus infection confirming their importance in antiviral response. Key immune response genes especially those encoding antiviral receptors such as *TLRs* and *RLRs* displayed strong association between variation in regulatory region and herpesvirus-caused mortality, suggesting they may confer resistance through transcriptional modulation. These results point to previously undescribed genetic mechanisms for disease resistance at different developmental stages and provide candidate polymorphisms and genes that are valuable for understanding antiviral immune responses and breeding for herpesvirus resistance.

## Introduction

Diseases of marine organisms, exacerbated by climate change and other human activities, are becoming more frequent and severe (1–3). Viruses, which account for 94% of the nucleic-acid-containing particles in the ocean and are a major cause of disease and mass mortality of diverse marine organisms (4). Molluscs are a major group of marine animals, many of which support important fishery and aquaculture industries (5, 6). Disease and mass mortalities have seriously affected molluscan aquaculture and led to immeasurable economic losses (1). As invertebrates, molluscs lack adaptive immunity and rely on an innate immune system for defense against pathogens (7). Understanding how molluscs without adaptive immunity cope with diverse viruses is of fundamental interest to immunology and evolutionary biology.

The Pacific oyster, *Crassostrea gigas*, is a marine bivalve that supports major aquaculture industries worldwide (8). The Pacific oyster is highly susceptible to the Ostreid herpesvirus 1 (OsHV-1) (9, 10), variants of which also cause infection and mass mortality of other marine bivalves such as scallops and clams (11–14). Mass mortalities of Pacific oysters caused by OsHV-1 have severely impacted global oyster production (1, 15–20). The sustainable development of the oyster industry is dependent on genetic improvement of cultured stocks particularly in disease resistance. Depending on the genetic architecture, disease resistance in oysters can be achieved by selective breeding quickly as in the cases of resistance to *Haplosporidium nelsoni* and *Roseovarius crassostreae* in the Eastern oyster *Crassostrea virginica*, or more slowly as in the case of Eastern oyster’s resistance to *Perkinsus marinus* (21, 22). Investigating the genetic architecture of OsHV-1 resistance is not only essential for genetic improvement of cultured stocks, but also important for our understanding of genes and variations that are critical in antiviral defense and adaptation in molluscs that lack adaptive immunity.

Genetic and molecular studies have been performed to understand and improve OsHV-1 resistance in the Pacific oyster, where vaccination strategies cannot be applied. Heritability values were estimated ranging from 0.078 to 0.63 (23–26) and varying among different developmental stages (24, 26). Breeding for OsHV-1 resistance has been conducted including four-generations of selection of families (24, 27), evaluation of triploids (28) and OsHV-1 variants (29). A genome-wide association study in the Pacific oyster using a SNP array identified a significant QTL in a region of linkage group 6 for OsHV-1 resistance (23).

Transcriptomic and proteomic studies have been conducted to understand host response to OsHV-1 and identified key genes and pathways involved in antiviral immune responses (10, 30–32). Genes and pathways that were significantly upregulated by OsHV-1 infection included *Toll-like* receptors signaling pathways (e.g., *TLR*, *MyD88*) (33, 34), *RIG-I-like* receptors signaling pathway (e.g., *RLR*, interferon induced factors, *IKKs*, and *cGAS* (10, 35–37), JAK/STAT signaling pathway (e.g., *STAT* and *SOCS2*), and antiviral immune effectors such as *antimicrobial peptides* (*AMPs*), *Viperin*, and *SAMHD-1* (38, 39). Genes and proteins involved in apoptosis regulation (e.g., *TNF*, *IAP*, and *caspase*) and autophagy (e.g., *ATG1*, *ATG8*/*LC3*, and *BECN1*) were activated by OsHV-1 infection (40). Proteins that show significant changes after OsHV-1 infection are involved in metabolic pathways such as host cytoskeleton, DNA replication and protein modification (30, 32). Genes involved in oxidation were upregulated, while genes involved in anti-oxidation were downregulated by OsHV-1 creating an oxidative burst that may be important for the destruction of viral components but also contribute to oyster mortality (10). Taken together, molecular investigations revealed a strong and complex antiviral response to OsHV-1 that involves many canonical innate immune response genes and pathways.

In this study, we investigated the genetic architecture of OsHV-1 resistance in Pacific oyster larvae and adults by identifying single-nucleotide polymorphism (SNPs), genomic regions and genes associated with mortalities caused by OsHV-1 using pooled whole-genome resequencing (41). We show that antiviral response or resistance in larvae and adults involves different sets of genes or differentiated members of expanded gene families. Key immune response genes were upregulated by OsHV-1 infection, including antiviral receptors such as *TLRs* and *RLRs* identified in previous studies, whose variation in promoter regions showed a strong association with mortality, indicating that resistance may be conferred through transcriptional regulation. Therefore, this study revealed previously undescribed genetic mechanisms for OsHV-1 resistance, and provided candidate polymorphisms that may be valuable for our understanding and improvement of OsHV-1 resistance.

## Materials and methods

We identified SNPs, genomic regions and genes associated with mortalities caused by OsHV-1 through pooled whole-genome resequencing of larval samples collected before and after mortalities, and of dead and surviving adult oysters from a challenge experiment. These SNPs, genomic regions and genes were regarded as mortality-associated or associated with OsHV-1 resistance.

### Samples collection and pooled sequencing

**Figure 1:**
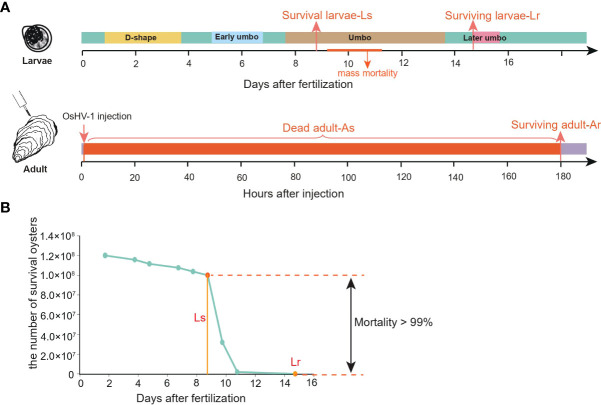
The Pacific oyster response to OsHV-1μVar infection in both larvae and adults. **(A)** schematic diagram of experimental design. Ls, larval susceptible population; Lr, larval resistant population; As, adult susceptible population; Ar, adult resistant population. **(B)** cumulative survival of oyster larvae (survival number) and mortality rates of pooled sequenced populations in larvae.

The Pacific oyster larvae used in this study were from an experimental cross of 1 male and 51 females from one family produced at the Qingdao Laodong Mariculture Breeding Company. A mass mortality of over 99% occurred during a short period at umbo development stage, which was caused by OsHV-1 (35). There was no known history of OsHV-1 infection in this population. We collected larvae before and after the mass mortality event as larval susceptible population (Ls) and larval resistant population (Lr), respectively, for whole-genome resequencing (**Figure 1**). Notwithstanding, the Ls population contained some Lr larvae, but proportionally neglectable because of the high mortality rate of over 99%. A large number of larvae (>10,000) were sampled to increase the accuracy of the pooled sequencing. OsHV-1 virus was first diagnosed by conventional PCR with OsHV-1 specific primers in dead larvae (42). A real-time PCR assay was further performed to detect OsHV-1 according to Pepin JF et al. (43), indicating the virus load of larvae reached the copy number of 7.5x10^5^/ng DNA around 9 days after fertilization (**Figure 1B**). We also estimated relative virus load before and after the larval mortality by sequence read mapping ratio using the following formula:


$$Viral\;mapping\;ratio=\log_{10}\frac{Reads\;mapped\;to\;the\;reference\;genome\;of\;virus\;(OsHV-1)\;\times \;10^{8}}{total\;sequenced\;reads}+1$$

The viral mapping ratio was about 4.4 before and 1.6 after the mortality event, and the acute and specific peak is positively correlated with larva mortality (**Figure S1**). DNA was extracted from Ls and Lr samples using extraction kit (Omega, USA).

Adult Pacific oysters used in this study (~18-month old, may also be referred as juveniles) were from an OsHV-1μVar (instead of OsHV-1) challenge experiment conducted at University of Caen, France by our team (10). Adult hatchery-produced oysters, not intentionally selected for OsHV-1μVar resistance, were obtained from a rack and bag system near Cricqueville-en-Bessin, on the coast of Lower Normandy (France), a location at least 4 km from any known oyster-growing area and established as a site where experimental oysters could be kept free of OsHV-1 µVar infections. Briefly, hatchery-produced oysters were injected with 1.5 ×10^9^ viral genomics units OsHV-1μVar. As determined with qPCR, only 1 VGU (viral genome unit) ng^-1^ of DNA extracted from the oysters was detected at Time 0, which increased to 2 x 10^2^, 1.9 x 10^4^, 2.9 x 10^4^ and 7.3 x 10^4^ VGUs ng^-1^ DNA at 6, 12, 24 and 48 h post-injection, respectively, compared with the 1 to 140 VGU ng^-1^ of DNA observed in unchallenged controls throughout the experiment (10). The 7.3 x 10^4^-fold increase in viral DNA load and its correlation with mortality suggest that the challenge was effective. The viral load of dead juvenile individuals ranged from 4.46 to 5.65×10^4^ VGUs ng^-1^ (10). Mortality began 20 hours after injection and reached up to 74% by 180 hours after injection (**Figure 1**). One hundred dead and 100 survivors were collected as adult susceptible (As) and adult resistant (Ar) populations, respectively. Genomic DNA was extracted from mantle and/or gill of each oyster on an EpMotion5075 automated pipetting system (Eppendorf^®^) with the Nucleospin 8 Blood kit (Macherey Nagel^®^) according to manufacturer’s protocol. Quality and quantity of template DNA was estimated on a Nanodrop 2000 spectrophotometer (Thermoscientific^®^), and equal amount of DNA from each individual was pooled to create two pools for sequencing.

Sequencing libraries were generated *via* adapter ligation, DNA cluster preparation, and subjected to 100 bp paired-end sequencing on an Illumina Solexa platform. Sequencing depth of each library was about 60 × for larvae and 45 × for adult populations. Resequencing was conducted by Beijing Genomics Institute Co., Ltd in Shenzhen, China.

### Data processing, mapping, and SNP calling

Clean data were obtained by trimming primer dimers and adaptors, discarding low-quality bases (Q20 as cutoff). Burrows-Wheeler Alignment Tool (BWA) software (http://bio-bwa.sourceforge.net/bwa.s-html) (44) was used to map the clean data to the Pacific oyster genome v9 (45). Duplicated reads generated by PCR amplification were removed. SAMtools (http://samtools.sourceforge.net/samtools.-shtml) (46), Picard (http://broadinstitute.github.io/picard/), and BCFtools (46) were used for SNP calling. Ambiguously mapped reads and reads with low depth were removed. The major filter parameters are: SNPs with sequencing depth no less than 10 and no more than 150, RMS >= 20, Qual >= 20, no indel within 15 bp of SNPs.

### Identification of SNPs, regions and genes associated with OsHV-1 resistance

SNPs with large difference in allele frequencies between the susceptible and resistant populations were extracted for both larval and adult samples. SNPs with major allele frequency differing no less than 0.5 were identified as associated with OsHV-1 or OsHV-1μVar susceptibility or resistance in this study. Genes in the 100 Kb upstream and downstream flanking regions were defined as associated genes.

To further explore genomic regions associated with oyster mortality, we estimated fixation index *F_ST_*. Based on our pooled resequencing data, we refer to the 2009 study (47) by Kent E. Holsinger et al. and choose to use *F_ST_* to describe a measure of allele frequency differences between populations. We defined *F_ST_* as (Molecular Population Genetics 1st Edition: by Matthew W. Hahn (Author)):


$$F_{ST}=\frac{\sigma_{s}^{2}}{\sigma_{T}^{2}}=\frac{\sigma_{s}^{2}}{\bar{p}(1-\bar{p})}$$

$\sigma_{s}^{2}$ represents the variance in allele frequency among populations; $\sigma_{T}^{2}$ represents the variance of the allelic state in the total population; $\bar{p}\;$ is the average frequency of the allele in the total population; $\bar{p}(1-\bar{p})$ is the variance in the allelic state for an allele chosen randomly from the entire population, so it can be regarded as a measure of genetic diversity in the entire population (47).

We estimated *F_ST_* in the sliding window of 20Kb. We first calculated the *F_ST_* values of four bases at each site, and took the largest absolute value among the four values as the final *F_ST_* value of the site. The mean *F_ST_* is the ratio of the total *F_ST_* of the window to the length of the window, which is used to sort and select the top 5% values for the regions under selection or related to disease/mortalities.

To detect large genomic regions in oyster that had experienced selection by the mortality, we mapped all the disease associated SNPs, regions, and genes onto the chromosomes of the genome. Ragtag (version=1.1.1) (48) was first used to assemble the v9 reference genome into chromosomes based on VN1 version information (GCA_011032805.1 (49)). The positions of the associated SNPs, regions, and genes were converted to the new chromosomal assembly through custom Python script. Circos software (version=0.69-9) (50) was used to draw the circle diagram.

### Transcriptome and transcription factor binding site analysis

To study the relationship between the transcription of immune response genes and genes associated with OsHV-1 resistance identified in this study, we downloaded and analyzed larval (33) and adult (10) transcriptomes under OsHV-1 infection that were obtained from the same challenge experiments (with the same larval and adult populations) as this study. Briefly, filtered sequencing reads were mapped to the oyster genomes by Tophat2 software (v2.1.1 (51)). Gene expression levels were calculated by fragments per kilobase of exon per million fragments mapped (FPKM) using HT-seq (52). The differentially expressed genes (DEGs) were identified with the edgeR tool (53) of the R programming language with the threshold value |log2FC| ≥ 1.5 (multiple of fold change, FC: difference) and FDR ≤ 0.05. In addition, the immune geneset used for comparison were obtained from Zhang L. et. al., 2015 (33).

The gene ontology (GO) and KEGG enrichment analyses were conducted with TBtools software (54) with the threshold value *p-value* ≤ 0.05. Fisher’s LSD and χ^2^ test was used to test significant enrichment when the number of genes in a GO term was< 5 and ≥ 5, respectively.

Transcription factor binding sites of upstream and downstream 300 bp of SNPs were predicted using an online transcription factor-binding site database, TRANSFAC *via* the PATCH™ 1.0 platform (http://gene-regulation.com/cgi-bin/pub/programs/patch/-bin/patch.cgi) with default parameters.

## Results

### Resequencing and SNP calling in larval samples

From the two larval pools collected before (Ls) and after (Lr) the mass mortality caused by OsHV-1 (**Figure 1A**), we generated 94 Gb sequences which, after filtering and mapping, covered 92.3% of the genome at average depth of 60-fold in both samples (**Table S1**). Depth analysis showed that the base depth distribution was similar to that of Poisson distribution in both samples (**Figure S2**).

After quality filtration, a total of 2,346,155 (4.75 SNPs/Kb) and 2,226,998 (4.52 SNPs/Kb) SNPs were identified in the Ls and Lr populations, respectively (**Table 1**). Among them, approximately 1.57 Mb and 1.49 Mb SNPs were transitions (variation between nucleotides of the same class, such as A/G and C/T), and 1.51 Mb and 1.44 Mb SNPs were transversions (variation between nucleotides of different classes, such as A/C, A/T, C/G and G/T) in Ls and Lr populations, respectively, corresponding to a Ts/Tv (transition/transversion) ratio of 1.04 in both populations (**Table 1**). In both populations, about 635 K (17.9%), 155 K (4.4%), 1,147 K (32.3%), 1,004 K (28.3%), and 609 K (17.2%) SNPs were located in downstream, exons, intergenic, introns, and upstream regions, respectively (**Table S2**). Among SNPs located in exons, about 61% were silent, 37% were non-synonymous, and 0.56% were nonsense (**Table 1**).

**Table 1:** Statistics of SNPs identified in the larval and adult populations.

Samples	SNP numbers	SNP rate(/gene)	Functional class						Transitions	Transversions	Ts/Tv
			NON-SYNONYMOUS	%	NONSENSE	%	SILENT	%			
Ls	2,346,155	84	61,046	37.855	904	0.561	99,312	61.584	1,570,879	1,510,186	1.040
Lr	2,226,998	79	57,584	37.753	853	0.559	94,092	61.688	1,492,136	1,435,381	1.039
As	2,552,189	91	67,191	36.414	800	0.434	116,530	63.153	1,513,023	1,433,387	1.056
Ar	2,808,259	100	72,636	36.312	912	0.456	126,486	63.232	1,661,994	1,577,039	1.054

Ls, larval susceptible population; Lr, larval resistant population; As, adult susceptible population; and Ar, adult resistant populations.

### Genomic loci associated with OsHV-1 resistance

**Figure 2:**
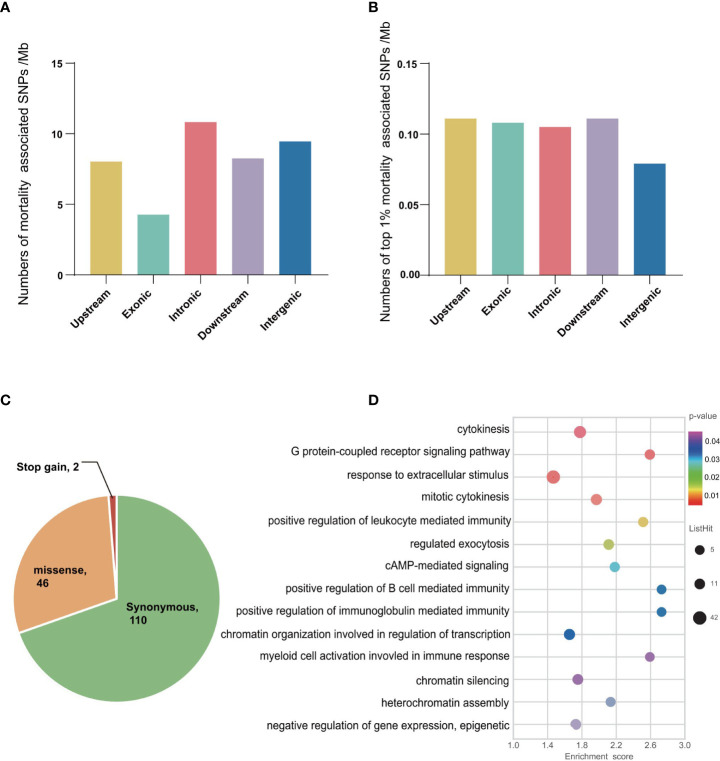
Description of mortality associated SNPs and genes in oyster larvae. **(A)** The number (per Mb) of mortality associated SNPs with base frequency not less than 0.5 between the susceptible and resistant larvae. **(B)** Statistics of the number (per Mb) of different classes of top 1% selective SNPs. **(C)** Characterization of mortality associated SNPs in exonic region. synonymous: causing no amino acid changes; non-synonymous: causing amino acid changes; stop gain: gain of stop codons. **(D)** Gene Ontology (GO) enrichment analyses of disease resistance associated genes in larvae. The 3,111 candidates were identified based on base frequency changes (no less than 0.5) and top 5% highest values of *F_ST_* analyses in the oyster larvae population.

To detect SNPs experienced selection by the mortality and associated with OsHV-1 resistance/susceptibility (also referred to as selective SNPs hereafter), we compared allele frequencies in larval samples collected before (Ls, mostly susceptible) and after (Lr, resistant) mortalities. Overall, we detected 117,613 and 5,271 SNPs with major allele frequency differing by at least 0.3 and 0.5 between the two larval populations, respectively (**Table 2**). Among the SNPs with allele frequency changes no less than 0.5, 217,158, 2057, 223 and 2630 were in upstream, exons, introns, downstream and intergenic regions, respectively. We further compared the number and frequency change level of the selective SNPs per Mb in different genomic regions (**Figures 2A, B**). Introns and intergenic regions have more selective SNPs than exons per Mb (**Figure 2A**). In addition, the number (42%) of genic selective SNPs (exons and introns, 227 Mb) was less than that (50%) in the intergenic regions (278 Mb). More SNPs with top 1% base frequency changes were located in genic region (especially introns) than intergenic regions, indicating that introns might plays an important role in virus resistance regulation. Among those selective or mortality-associated SNPs in coding regions, 110 SNPs were synonymous, and 46 were non-synonymous. We also found 2 pre-mature stop-codons in open reading frames, and no mutations from stop codons to amino acid codons (**Figure 2C**). Overall, 44% of selective SNPs in non-coding regions were not in the flanking regions around a gene. A total of 2,949 (56%) of selective SNPs were located in the genic or flanking regions around a gene.

**Table 2:** Number of mortality-associated SNPs and *F_ST_* outlier regions in larval and adult populations.

	Mortality-associated SNPs				Mortality-associated *F_ST_* regions			
	Allele Frequency Changes≥0.3	Allele Frequency Changes≥0.5	Rate of Allele Frequency Changes ≥0.5	Overlapped Genes	*F_ST_* Regions	The Length of Sliding Window	Top 5% *F_ST_* regions	Overlapped Genes
Larvae	117,613	5,271	3.95%	1,831	37,660	20kb	1,883	1,401
Adult	344,832	18,692	5.42%	3,683	566,294	1kb	28,314	1,401

To identify regions in oysters that experienced selection by the OsHV-1 caused larval mortality, we conducted selective sweep analysis with the *F_ST_* statistics. Of the 37,660 20-Kb windows screened, 1,883 regions spanning 23 Mb had *F_ST_* values among the highest 5%. In these regions, we detected 1401 genes as showing selection signal (**Table 2**).

### Candidate genes associated with OsHV-1 resistance in larvae

We identified candidate genes associated with OsHV-1 resistance by examining allele-frequency shifts and *F_ST_* signals. A total of 1,831 genes were identified by SNP allele-frequency shift analysis. The highest allele-frequency shift (0.77) was observed at a SNP at base 81,745 of scaffold 41,452, which was flanked by two *peripheral myelin protein 22* (*PMP22*) genes, with crucial roles in peripheral nervous system pathological processes (55–57). Since the nervous system is the main target tissue of oyster herpesvirus (57, 58), we speculate that SNP 81,745 might be associated with regulatory elements of *PMP22* and hence affect virus resistance. In addition, *BTG3 associated nuclear protein* (*BANP*) was found in a region with the highest *F_ST_* value in scaffold 448. *BANP* is known to negatively regulate the transcription of tumor protein p53 (59) and apoptosis (60). A *telomere-associated protein RIF1-like* gene, which promotes DNA repair (61), was located in a region with the second highest *F_ST_* value in scaffold 448. Analyses of those 1,831 genes (**Table S3**) revealed enrichment of GO terms for many biological processes including ‘G protein-coupled receptor signaling’, ‘cell division’, ‘electron transport chain’, ‘carbohydrate derivative metabolic processes’, ‘positive regulation of exocytosis’, and ‘regulation of lipase activity’. A total of 1,401 candidate genes were identified by the *F_ST_* distribution analysis where GO terms related to viral penetration into cells, envelope assembly and release were significantly enriched (**Table S3**), including ‘regulation of DNA replication’, ‘cytokinesis and cellular response to xenobiotic stimulus’, ‘vesicle fusion’, and ‘glycoprotein biosynthetic process’.

Together, allele-frequency changes (no less than 0.5) and *F_ST_* outlier (largest 5%) analyses produced a set of 3,111 candidate genes that were regarded as under selection by mortalities or associated with OsHV-1 resistance. Analysis of those genes revealed significant enrichment of GO terms related to immune response (**Figure 2D** and **Table S3**) including ‘regulation of leukocyte mediated immunity’, ‘regulation of B cell mediated immunity’, ‘positive regulation of immunoglobulin mediated immune response’, and ‘myeloid cell activation’. The following enriched GO terms may be related to the assembly and release of OsHV-1 viruses: ‘negative regulation of gene expression and chromatin organization involved in negative regulation of transcription’, ‘exocytosis and regulation of regulated secretory pathway’. In addition, 121 candidate genes were implicated by both allele-frequency shift and *F_ST_* outlier analyses, including ten genes annotated as *TRIM* genes, which are involved in the PRR signaling pathway and play important roles in innate immune.

### Genomic loci and regions associated herpesvirus resistance in adults

To identify genomic loci and regions related to herpesvirus resistance in adults, we performed whole-genome resequencing of pooled samples of dead and survived oysters from an OsHV-1μVar challenge experiment. We generated ~67 Gb high quality sequences which, after filtering and mapping, led to an average mapping depth of 45-fold (**Table S1**). Alignment and SNP calling identified 2.55 and 2.81 million SNPs in the dead/susceptible (As) and survivor/resistant (Ar) populations, respectively (**Table 1**). A total of 18,692 SNPs showed significant allele-frequency differences between As and Ar populations, involving 3,683 genes (**Table 2**). In addition, we found that the number of selective SNPs per Mb was greatest in intronic and intergenic regions than that in exons (**Figure S4A**). The SNP at base 19,753 of scaffold 37,760, located in the first intron of an *epidermal growth factor* (*EGF*) gene, showed the highest 0.92 allele-frequency shift. The *EGF* superfamily plays an important role in regulating growth, proliferation, and differentiation of the numerous cell types, which might also be involved in herpesvirus resistance.

In addition, analysis of *F_ST_* between the two populations identified 28,314 regions in the top 5% of *F_ST_* distribution, covering 1,401 genes (**Table 2**), including a *sacsin-like* gene, a member of heat shock proteins (62–66), located in a region of scaffold 942 with the highest *F_ST_* value. In combination, the two methods identified 4,863 candidate genes associated with OsHV-1μVar resistance in adults. Among these genes, the GO terms ‘regulation of *Toll-like* receptor signaling pathway’, a canonical immune response pathway, was significantly enriched (**Table S4**). Enriched pathway and functional terms also included ‘regulation of production of molecular mediator of immune response’, ‘protein localization to Golgi apparatus’, ‘translational initiation’ and ‘exosomal secretion’. In addition, GO terms related to stress response was also significantly enriched including ‘regulation of JNK cascade’, ‘positive regulation of JUN kinase activity’, and ‘activation of MAPKK activity’. These observations indicate that genetic variation in genes related to innate immune, virus assembly, and stress response are important for herpesvirus resistance in adults.

**Figure 3:**
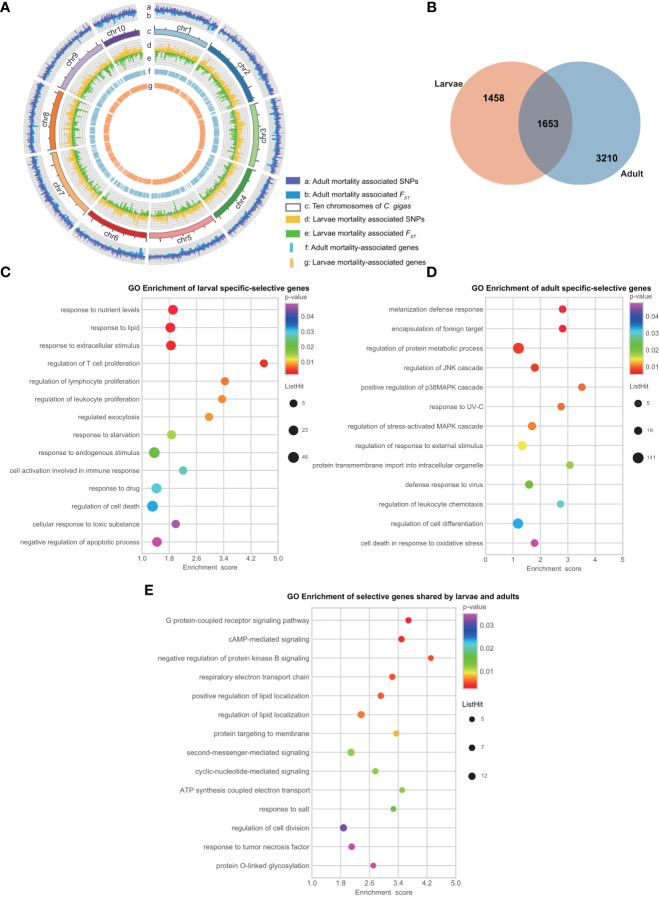
Comparative analysis of genomic loci and genes associated with larval and adult disease resistance. **(A)** Circos analysis of mortality associated genomic loci and regions. The allele frequency of the locus is proportional to the column height. From outside to inside (named a-g): the a and b layers represent the mortality associated SNP loci and *F_ST_* regions of the adult population; the c layer represents ten chromosomes of the oyster genome; the d and e layers represent the mortality associated SNP loci and *F_ST_* regions of the larval population; the f and g layers represent mortality associated genes in the adult and larval populations. **(B)** Venn diagram showed the difference of disease selected gene sets between larvae and adults based on base frequency and *F_ST_* analyses. **(C–E)** Gene Ontology (GO) enrichment analyses of 1,458 larvae specific, 3,210 adult specific, and 1,653 larva-adult shared selective genes.

To determine if those candidate genes from adults were the same as those identified from larvae, we mapped the differential SNP and *F_ST_* sites at the chromosome level with a circular map (**Figure 3A**). Mortality-associated sites were distributed on all chromosomes in both larvae and adult populations with few concurrent peaks, suggesting OsHV-1 resistance in larvae and adults involve different genomic regions or genes. In addition, 1,234 (23%) selective SNPs identified with allele-frequency shifts were located in regions with the top 5% *F_ST_* value in larvae, and 7,404 (40%) in adults. In terms of genes under selection by the mortality, 1,653 genes were shared by larvae and adults, while 1,458 and 3,210 genes were specific to larvae and adult oysters, respectively (**Figure 3B** and **Table 3**). Our results show that there are fewer mortality-related genes in larvae than in adults (**Figure 3B**). The larval and adult populations were from China and France, respectively. Differences in genetic background, OsHV-1 or OsHV-1μVar virulence and exposure history may contribute to the difference in the number of selective genes observed. SNP polymorphism of the larval population (2.69% before and 2.74% after mortality) was similar to that of the adult population (2.60% for dead and 2.55% for survivors). It is possible that the difference in the number of genes under selection between larvae and adults is caused by developmental differences in immune response, as the types of genes identified were also different.

**Table 3:** Selected mortality associated genes in Pacific oyster larval and adult populations identified by allele-frequency shift and fixation statistics (FST).

	Gene	Scaffold	Gene_Start	Gene_End	Gene_Annotation	Larvae		Adult	
						*F_ST_*	Max (Allele Frequency shift)	*F_ST_*	Max (Allele Frequency shift)
larvae-specific	OYG_10002102	scaffold36732	18064	24631	*C1qDC*	0.08	0.45	–	–
	OYG_10007060	scaffold1758	11397	20838	*IFRD1*	0.08	–	–	–
	OYG_10021170	scaffold4	155258	160967	*IRF1*	0.06	–	–	–
	OYG_10023843	scaffold406	351742	352809	*HIP*	0.01	–	–	–
adult-specific	OYG_10001779	scaffold598	6664	7095	*C1qDC*	–	–	–	0.53
	OYG_10002498	scaffold37790	1900	11693	*C1qDC*	–	–	0.03	0.54
	OYG_10002780	scaffold38448	38584	39306	*TRIM3*	–	–	0.01	0.61
	OYG_10001483	scaffold74	22091	23733	*C1qDC*	0.007	–	0.06	0.48
	OYG_10003270	scaffold1598	18526	29890	*IRF8*	0.043	–	–	0.61
	OYG_10014035	scaffold983	353704	360265	*RLR*	0.008	–	0.09	0.65
	OYG_10003004	scaffold38884	44613	50381	*C-type lectin A*	–	–	–	0.71
	OYG_10003934	scaffold40224	80124	80803	*C1qDC*	–	–	–	0.54
shared by larvae and adults	OYG_10004092	scaffold1558	2192	4793	*SOD*	0	0.55	–	0.62
	OYG_10004837	scaffold41296	5403	5996	*NF-kappa B*	0	0.53	–	0.46
	OYG_10004826	scaffold178	58024	92952	*CD109*	0.1	0.51	–	0.73
	OYG_10004656	scaffold41064	24043	43070	*TBK1*	0.059	0.51	0.14	0.61
	OYG_10005133	scaffold41522	11479	33348	*IRF*	0.154	0.58	0.38	0.52
	OYG_10005135	scaffold41522	44800	58091	*C1qDC*	0.058	0.58	0.16	0.52
	OYG_10005421	scaffold1256	20370	32211	*Toll-like*	0.06	0.53	–	0.58

The genes shared by larvae and adults included those coding for *Toll-like* receptor, tripartite motif-containing (*TRIM*), *NF-kappa B*, Extracellular superoxide dismutase (*SODE*) and immunoglobulin, all key members of antiviral immune signal pathway. Although large numbers of the mortality-associated genes were specific to larval or adult populations, some of them were paralogs and potentially have similar functions. For example, *C1qDC* (OYG_10002102) showed significant association in larvae, whereas a member of the same gene family *C1qDC* (OYG_10002498) was associated with OsHV-1 μVar resistance in adult oysters. The findings of relatively fewer genes (1,653 genes) shared between larvae and adults, and the involvement of divergent paralogs with similar functions support the hypothesis that herpesvirus response and resistance has diverged somewhat between different developmental stages. Again, virus strain difference may also be a contributing factor.

Despite the variation in mortality-associated genes between larvae and adults, the most prevalent or highly enriched genes in both populations included those involved in immune response, virus assembly and release (**Figures 3C–E**; **Table 3** and **Tables S3, S4, S5**). For example, GO terms related to T cells regulation, white blood cells, lymphocytes, and apoptosis were enriched (**Figure 3C**). The immune response genes under those GO terms include *tyrosine-protein kinase JAK2, polycomb complex protein BMI-1-A, interferon regulatory factor 1-like*, and *Toll-like receptors*. In adults, GO terms related to encapsulation of foreign target, regulation of JNK cascade and p38 MAPK cascade were also enriched (**Figure 3D**).

### Innate immune genes associated with OsHV-1 caused mortalities

**Figure 4:**
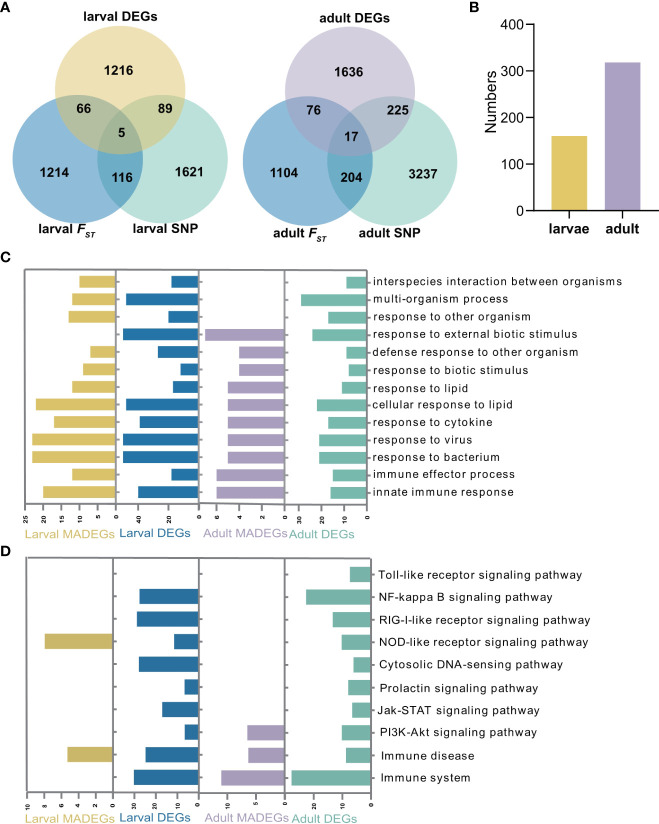
Analysis of differentially expressed and mortality-associated genes. **(A)** Venn diagrams of differentially expressed and mortality associated genes in larval and adult populations, respectively. DEG: differential expressed genes under virus infection. **(B)** The number of mortality-associated DEGs (MADEG) in larvae and adult populations, respectively. **(C)** The Gene Ontology (GO) and **(D)** Kyoto Encyclopaedia of Genes and Genomes (KEGG) enrichment analyses of the MADEGs in larvae and adult populations, and their comparisons with all DEGs.

To assess whether the mortality-associated genes are involved in immune response to herpesvirus infection, we analyzed transcriptome data for the same larval (33) and adult (10) populations subjected to OsHV-1 infections collected in previous studies. Our analyses identified 1,376 and 1,954 differentially expressed genes (DEGs) in larvae and adult oysters, respectively (**Figure 4A**). In larvae, 160 (5.2%) mortality-associated genes were differentially expressed during OsHV-1 infection, whereas 318 (6.5%) mortality-associated genes were differentially expressed in adults under OsHV-1 μVar infection (**Figure 4B**). Analyses of the mortality-associated DEGs in the larvae and adult oysters revealed enrichment of similar GO terms related to ‘response to virus’, ‘response to cytokine’ ‘immune effector process’ (**Figure 4C**), but somewhat different KEGG pathways (**Figure 4D**). In larvae, pathways ‘PRR signaling pathway’ and ‘development and regeneration’ were significantly enriched (**Table S7**). In adults, pathways related to stress response ‘*PI3K*-*Akt* signaling pathway’ (*p* = 0.01), innate immune related pathways ‘immune system’, ‘complement and coagulation cascades’ and ‘immune disease’, and cellular processes related pathway ‘phagosome’ were also significantly enriched (**Table S7**). In both larvae and adults, immune pathways were enriched in mortality-associated DEG, indicating immune response genes were more likely associated with OsHV-1 caused mortalities.

**Figure 5:**
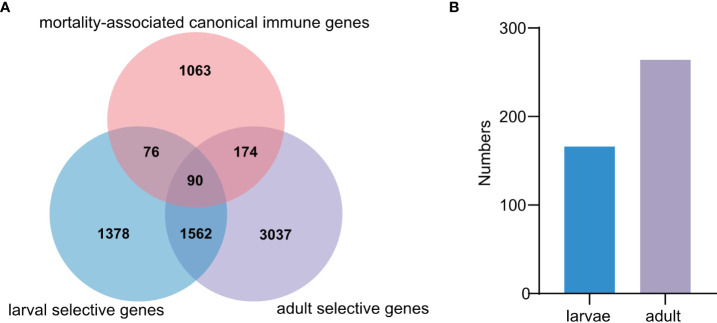
Comparative analysis of mortality-associated and canonical immune genes. **(A)** The Venn diagram represents the differences of mortality-associated and canonical immune genes in larvae and adult populations, respectively. **(B)** The number of mortality-associated genes annotated as canonical immune genes in larval and adult populations, respectively.

To determine how many immune genes are associated with herpesvirus caused mortalities, 1,403 canonical innate immune genes, belonging to 61 families and 4 innate immune pathways (33), were analyzed, among which 340 genes (24.2%) were associated with OsHV-1 caused mortalities (**Figure 5A**). Specifically, 166 and 264 mortality-associated genes identified in larvae and adults, respectively, were canonical innate immune genes (**Figure 5B**). Among them, 90 were mostly immune receptors associated with OsHV-1 caused mortalities in both larvae and adults, including those from the Toll-like signaling pathway (seven *TLR receptors*, *ECSIT*, *TBK*, *IKK*, *IRF, NF-kappa B*, and *Interleukin-like*) and other immune receptors (20 *C1qDCs*, 16 *C-type lectins*, five *SRCRs*, eight *FBGDCs*, and one *Alpha-2-macroglobulin like*), and effectors *(SODs* and *Serine/threonine-protein kinase*) (**Table S8**).

Analysis of mortality-associated immune genes from larvae and adults revealed the enrichment of two KEGG pathways central to immune signaling, ‘*Toll-like receptor* signaling pathway’ and ‘*RIG-I-like receptor* signaling pathway’ (**Table S9** and **Table S10**). Other enriched pathways included viral proliferation related ‘*Ras* signaling pathway’, proinflammation related ‘*IL-17* signaling pathway’, and viral multiplication related ‘proteoglycans and glycosaminoglycan binding proteins’.

### Antiviral immune receptors associated with mortality

The oyster’s innate immune system plays a major role in oyster resistance to pathogens through pattern recognition receptors (PRRs). We found the receptors *TLRs* and *RLRs* were strongly associated with virus infection based on the presented results including the number and frequency change level of selective SNPs nearby (genic, upstream 50Kb, and downstream 50Kb). We sorted all the 28,027 genes in the oyster genome based on the number of selective SNPs nearby and found 12 *TLRs* rank in the top 5%. Meanwhile, one *RLR* genes have five selective SNPs nearby, which ranks top 2% of all the oyster genes. Meanwhile, we also sorted all the 28,027 genes based on the maximum frequency change level of selective SNPs nearby. Virus resistance associated *TLRs* and *RLRs* in this study fall in the top 10% of the all the genes.

**Figure 6:**
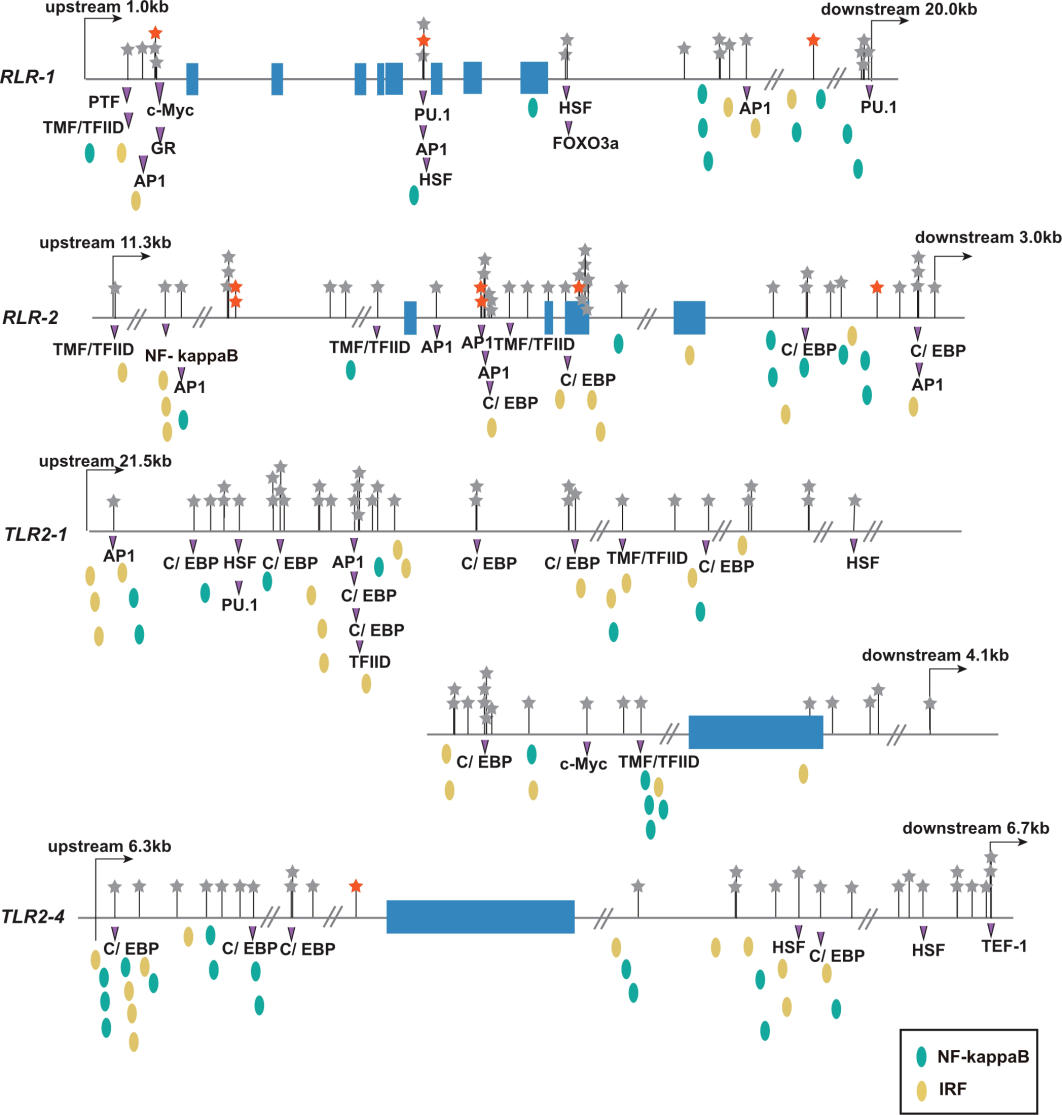
Distribution of polymorphism and transcriptional factor binding sites at four pattern recognition receptors (*RLR-1*, *RLR-2*, *TLR2-1*, and *TLR2-4*) associated with OsHV-1 caused mortality. The blue rectangles are exons. The stars represent SNPs, and the red stars indicate SNPs whose allele frequency changes is no less than 0.5. The purple triangle represents the predicted transcription factor binding sites that covers SNPs. The canonical antiviral transcription factor binding sites IRF and NF-kappa B near SNPs are marked with yellow and green ellipses, respectively.

Given that the antiviral pattern recognition receptor genes *TLRs* and *RLRs* showed significant allele-frequency differences between susceptible and resistant populations, we conducted detailed analysis of these receptors. Among the three *TLRs* and nine *RLRs* responding transcriptionally to OsHV-1 (33, 35), two *TLRs* and two *RLRs* were associated with OsHV-1 caused mortalities in both larvae and adults in this study (**Table S11**). To explore the potential function of mortality-associated sites, we predicted transcription factor binding sites around the *TLRs* and *RLRs*. Transcription factor binding sites were densely populated upstream and downstream of the four PPR genes, which were also where most of mortality-associated SNPs located (**Figure 6**). Few SNPs were located in exons, and the only one in *TLR2-1* was non-synonymous. The concurrence of mortality-associated SNPs with transcription factor binding sites suggests that these SNPs may affect transcription factor binding and regulate the expression of antiviral immune receptors (**Figure 6**). The predicted transcription factors included that for IRF, NF-kappa B, HSF, C/EBP, and AP-1, all important for immune and stress responses. Overall, these results suggest that PRRs are critical for host-defense against viral infection, and variations at transcription factor binding sites may determine resistance to viral infections.

## Discussion

Massive mortality outbreaks affecting Pacific oyster larvae and adults are often caused by OsHV-1 (including μVar). Mortality caused by OsHV-1 infection is rapid and heavy, but some oysters can survive with greatly reduced virus load, indicating that some oysters may be resistant (10). While studies have examined host responses to OsHV-1 in juvenile or adult oysters (10, 23, 30, 67, 68) and selected for OsHV-1 resistance (37, 69), the genetic architecture of OsHV-1 resistance is largely unknown especially for larvae. In this study, we took advantage of larval samples collected just before and after a mass mortality event where over 99% of the larvae died due to OsHV-1 infection (**Figure 1**). We conducted pooled whole-genome sequencing of these samples to identify polymorphisms and genes associated with OsHV-1 caused mortalities. We further compared mortality associated genes from larvae with that identified from dead and surviving adults from an OsHV-1 (μVar) challenge experiment. Our analyses identified many immune response genes associated with OsHV-1 resistance, especially two *TLRs* and two *RLRs*. Some of the genes identified in this study may not be canonical immune response genes, and most of variants associated with resistance appeared to be intergenic and anonymous. Nevertheless, this study provides a set of candidate genes and polymorphisms that are valuable to our understanding of antiviral defense and the development of OsHV-1 resistant oysters for aquaculture. It should be noted that, despite the use of stringent thresholds and two different methods (allele-frequency changes and *F_ST_* outliers), the depth of pooled resequencing is limited, and the function of some of immune related genes is uncertain. Further studies may be needed to confirm some of the findings.

Several findings from our study are interesting and worth highlighting. First, genes associated with OsHV-1 resistance in larvae and adults are largely different. Overall, more mortality-associated genes were observed in adults than in larvae, but more genes related to antiviral defense and viral proliferation were implicated in larvae than in adults. These findings suggest that antiviral defense in larvae and adults may be different or involve different set of genes, although differences in genetic background, OsHV-1 strain and exposure history, and environmental conditions may also be partly responsible. Previous studies have shown that young juveniles are more susceptible to OsHV-1 infection than adults (70), and the difference may be due to a gradual maturation of immune system as oysters develop into adults, or due to the adults being selected by the virus during the larval stage. The fact that different sets of genes were implicated suggests that larvae and adults may differ in their antiviral defense, which is not surprising as they represent very different developmental stages. In some cases, different members of expanded gene families are implicated in larvae and adults, suggesting some differentiation or specialization in gene function. The differentiation or specialization of immune response genes in larvae and adults may be the consequence of the great expansion of immune/stress response genes observed in bivalve molluscs (33, 71–75). Duplicated gene members may diverge and become specialized in assuming the same or similar functions in different organs, at different developmental stages or under different environmental conditions (45, 76). The expansion and diversification of stress and immune related gene is considered central to the adaptation of marine bivalves.

Second, we found more (58% in larvae and 59% in adults) selective SNPs in non-genic regions compared to the proportion (3% in both larvae and adult) of SNPs in coding regions (exonic regions). This suggests that regulation of target genes is the main function of these selective SNPs, rather than altering protein structure. These selective SNPs in noncoding regions might be located in non-coding RNAs, enhancer, and promoters that regulate the expression of genes nearby or far away (77–79). Our finding is consistent with previous studies that non-coding region SNPs were important targets of selection (80, 81). Previous studies in oysters revealed many distant regulatory SNPs around heat-responsive genes in oysters (82), involving transcription factors, nuclear receptors, miRNAs and small nuclear RNAs (83). Future studies on non-coding variation with miRNA-seq, BS-seq, ATAC-seq, Histone marker CHIP-seq, are needed to illuminate the function of those intergenic selective signals.

Third, the identification of genes associated with OsHV-1 caused mortalities provided insights into antiviral defense mechanisms in the Pacific oyster. Among the mortality or resistance associated genes, genes of the *PI3K*-*Akt* signaling pathway were significantly enriched. Previous studies (84–86) have shown that the *PI3K* signaling pathway inhibits the transcription of pro-apoptosis genes, thereby reducing apoptosis, promoting cell survival or inhibiting host cell death caused by viral infection. We hypothesized that variation in genes of the *PI3K* pathway affects survival or OsHV-1 resistance through its regulation of host-cell apoptosis. This finding is consistent with the significant upregulation of 6 *inhibitors of apoptosis* in OsHV-1 infected Pacific oyster (10). The *PI3K* pathway genes were also associated with columnaris resistance in catfish (87), and *Porphyromonas gingivalis* could activate *PI3K* in mice (88). In addition, the mortality associated genesets from both larvae and adults included a gene coding for superoxide dismutases (*SOD*), where three SNPs nearby showed significantly changes in allele frequency. *SODs* are critical in protecting cells against damage caused by ROS (89–91). A qRT-PCR study has shown that this *SOD* gene showed lower mRNA expression in heavily than lightly OsHV-1 infected oysters (92).Another study in the Pacific oyster found that five *SOD* genes were down-regulated by OsHV-1 by an average of 34-fold, along with the down-regulation of other anti-oxidation genes and upregulation of oxidative genes (10). Variations in *SOD* may confer OsHV-1 resistance by regulating a robust oxidative burst is critical in the destruction of viral components but may also be harmful to the host. Another gene significantly associated with OsHV-1 mortality encodes *CD109* of the thioester-containing proteins superfamily, which plays important roles in activating T-cells and endothelial cells (93–95), affecting cell proliferation, cell death and other processes by negatively regulating transforming growth factor beta (*TGF-beta*) signaling pathway (96). The gene encoding CD109 was associated mortality from Ovine Johne’s disease in Turkish sheep (97). Therefore, variations near *CD109* may affect OsHV-1 resistance through its regulation of cell proliferation *via TGF-beta* signaling pathway, affecting OsHV-1 pathogenesis.

Although the 10 SNPs showing strongest association with survival and OsHV-1 load identified in a previous (23) did not show significant base frequency changes (≥ 0.5) in our study, many of those genes were in the mortality associated geneset from this study including four of the survival associated genes (*KPNA1*, *CASP*, *AP1AR* and *KIF6*) and four viral load associated genes (*FBN2*, *CARS*, *TNIK* and *SCARF2*). The differences in the results may be due to difference in OsHV-1 strain, genetic background of the oysters used and experimental conditions.

Fourth, our study suggests two canonical antiviral immune receptors *TLR* and *RLR* play important roles in herpesvirus resistance in the Pacific oyster. *TLRs* are important PRRs that function in pathogen recognition and immune response (98–100). Previous research has shown that the two selective *TLRs* identified in this study were significantly upregulated in response to OsHV-1µVar infection (10, 35, 101, 102). In addition to *TLRs*, retinoic acid-inducible gene I (*RIG-I*)-like receptors (*RLRs*) that are localized in the cytosol, are the main nucleic acid sensors for pathogen recognition critical for sensing viral nucleic acids (103, 104). Previous knockout studies in mice and mouse-derived cells established that *RLRs* are essential for antiviral defense and type I interferon induction in virus infection models (105, 106). Almost all *RLRs* were significantly up-regulated by OsHV-1μVar in the Pacific oyster (10, 33, 35), and two of them were associated with herpesvirus caused mortalities in both larvae and adults in this study. In addition, KEGG enrichment analysis indicates that *TLR* and *RLR* pathways were significantly enriched in DEGs in infected larvae and adults. Taken together, these evidences suggest that SNPs around *TLR* and *RLR* genes are closely related to OsHV-1 resistance in oysters. Importantly, most mortality-associated SNPs near the four PRR genes are found in regulatory regions rich in transcription binding sites of immune regulators such as IRF and NF-kappa B. This finding suggests that these polymorphisms may confer disease resistance through transcriptional regulation of PRRs and downstream immune signaling pathways. In the Eastern oysters, resistance to *Perkinsus marinus* is influenced by polymorphism in the promoter region of a serine protease inhibitor (107), and shell growth is regulated by both transcriptional modulation and functional polymorphism, two separate mechanisms that may act on different sets of genes (108).

In summary, pooled whole-genome resequencing of susceptible and resistant samples of larvae and adults provided insights into the genetics of herpesvirus resistance in the Pacific oyster. Our analyses identified a large number of loci and genes associated with herpesvirus caused mortality including canonical immune response genes related to antiviral response, many of which also showed significant transcriptional upregulation in herpesvirus infected oysters. We showed that antiviral defense may differ between larvae and adults as indicated by differences in mortality-associated genes observed. Polymorphisms in regulatory regions of two *TLRs* and two *RLRs* exhibited strong association with herpesvirus caused mortality, revealing the importance of PRRs and transcriptional regulation in effecting viral defense and resistance. Overall, this study provides previously undescribed genetic mechanisms for disease resistance at different developmental stages and a rich set of polymorphisms and genes that may be valuable for understanding antiviral immune response and breeding for herpesvirus resistance.

## Data availability statement

The datasets presented in this study can be found in online repositories. All sequencing data presented in the study were deposited in the National Center for Biotechnology Information (NCBI) Sequence Read Archive database, BioProject Accession Numbers PRJNA828432.

## Author contributions

Conceived and designed experiments: GZ, XMG, and LZ. Data analyses: SY, XDG, LZ, and XMG. Challenge experiment and sample collection: YH, AJ, XMG, FX, LZ, LL, and GZ. Contributed reagents/materials/computer resources: LZ, GZ, XMG, and LL. Wrote the paper: SY, LZ, and XMG. All authors read and approved the final manuscript.

## Funding

This research was supported by the Marine S&T Fund of Shandong Province for Pilot National Laboratory for Marine Science and Technology(Qingdao) No. 2022QNLM030004 to LZ, the Strategic Priority Research Program of the Chinese Academy of Sciences XDB42000000 to LZ, the National Natural Science Foundation of China 41976088 to LZ, and the Key Development Project of Centre for Ocean Mega-Research of Science, Chinese Academy of Science COMS2019R01 to LZ. The adult challenge experiment was supported by the “Conseil Régional de Basse-Normandie” and “Fonds Européen de Développement Régional” (PO FEDER 2007-2013; “Chaire d’excellence” project: “Prof. GUO Ximing: adaptabilité de l’huître creuse à son environnement”). XMG is supported in part by USDA-NIFA Hatch and NJAES Animal Health Project 1004475/NJ32920.

## Acknowledgments

We thank the high-performance computing center of Institute of Oceanology, CAS, for providing resources, the late Dr. Susan Ford for contributing to challenge experiments and Dr. Pascal Sourdaine for program support.

## Conflict of interest

The authors declare that the research was conducted in the absence of any commercial or financial relationships that could be construed as a potential conflict of interest.

## Publisher’s note

All claims expressed in this article are solely those of the authors and do not necessarily represent those of their affiliated organizations, or those of the publisher, the editors and the reviewers. Any product that may be evaluated in this article, or claim that may be made by its manufacturer, is not guaranteed or endorsed by the publisher.

## Supplementary material

Supplementary Figure 1 Response of oyster larvae to OsHV-1 infection. Ratio of RNA-seq reads mapping to the OsHV-1 genome and the mortality of oyster larvae are shown (35) (the data refers to Huang et al,. 2017).

Supplementary Figure 2 Depth distribution of the four pooled sequenced samples. Depth was calculated for each base from the aligned reads. Reads with multiple alignment were assigned to one best-hit location. Sequencing depth exhibited a Poisson-like distribution on different samples, respectively.

Supplementary Figure 3 Box plots of the allele frequency changes of selective SNPs between different genomic regions (upstream, downstream, exons, introns, and intergenic) in larvae **(A)** and adults **(B)**.

Supplementary Figure 4 Description of mortality associated SNPs in oyster adults. **(A)** The number (per Mb) of mortality associated SNPs with base frequency not less than 0.5 between the susceptible and resistant adult. **(B)** Statistics of the number (per Mb) of different classes of top 1% selective SNPs.
