# Supplementary figures and images for: Pooled resequencing of larvae and adults reveals genomic variations associated with Ostreid herpesvirus 1 resistance in the Pacific oyster Crassostrea gigas
Source: Front Immunol. 2022 Aug 19;13:928628. doi: 10.3389/fimmu.2022.928628 (PMC9437489; doi:10.3389/fimmu.2022.928628)

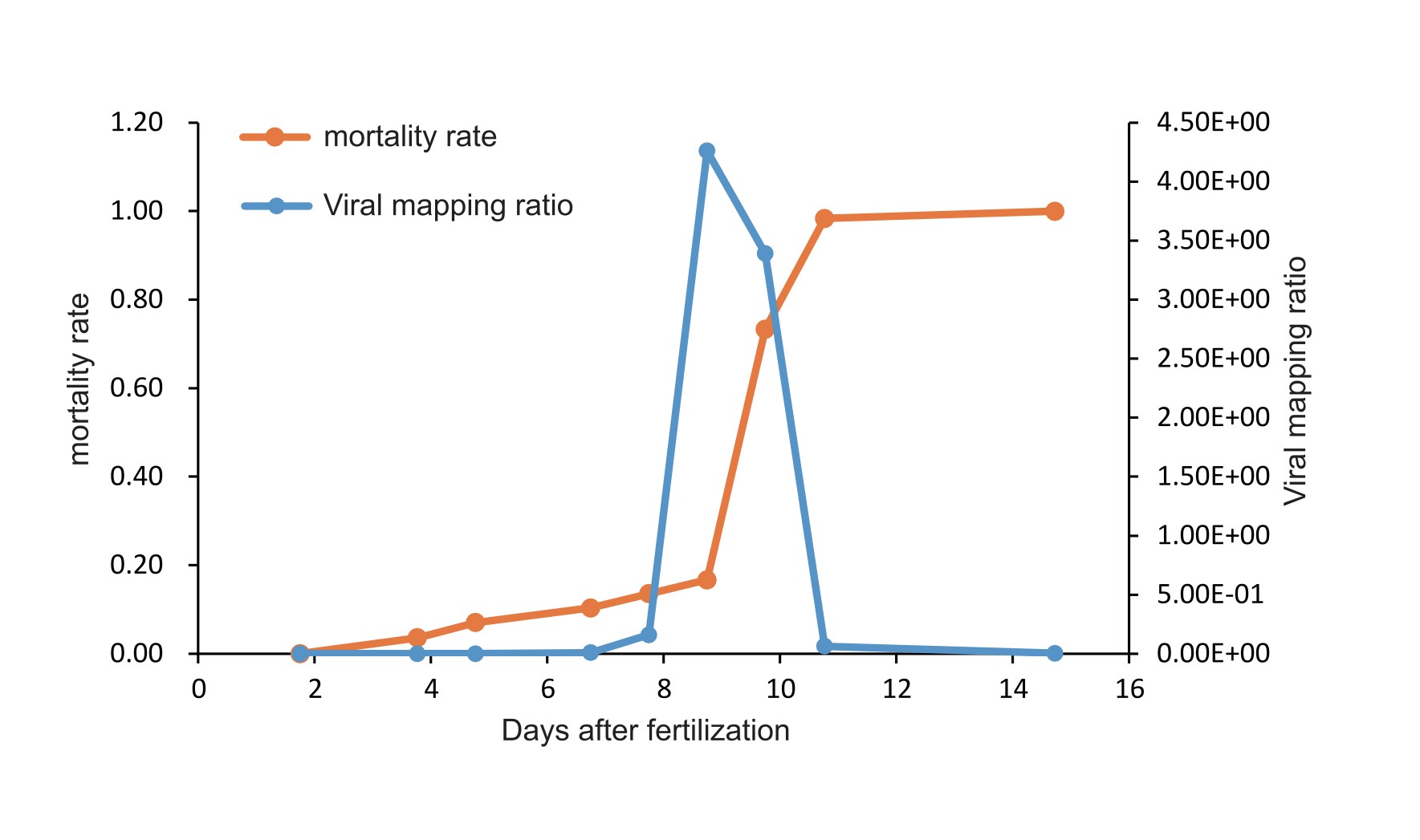

Supplement: Supplementary Figure 1 — Response of oyster larvae to OsHV-1 infection. Ratio of RNA-seq reads mapping to the OsHV-1 genome and the mortality of oyster larvae are shown (35) (the data refers to Huang et al,. 2017). [file DataSheet_1.zip › Supplementary Materials/Figure S1.jpg]

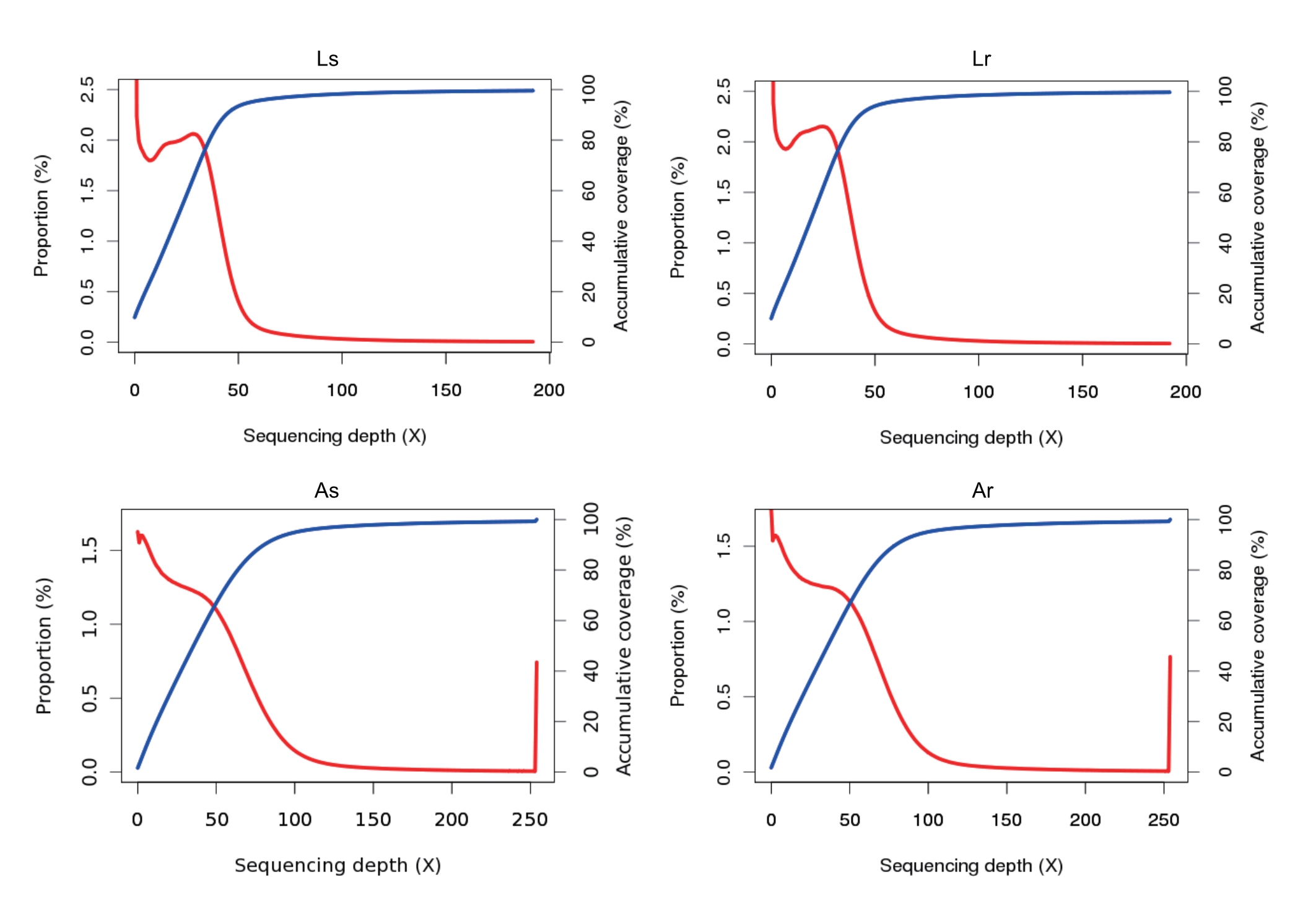

Supplement: Supplementary Figure 1 — Response of oyster larvae to OsHV-1 infection. Ratio of RNA-seq reads mapping to the OsHV-1 genome and the mortality of oyster larvae are shown (35) (the data refers to Huang et al,. 2017). [file DataSheet_1.zip › Supplementary Materials/Figure S2.jpg]

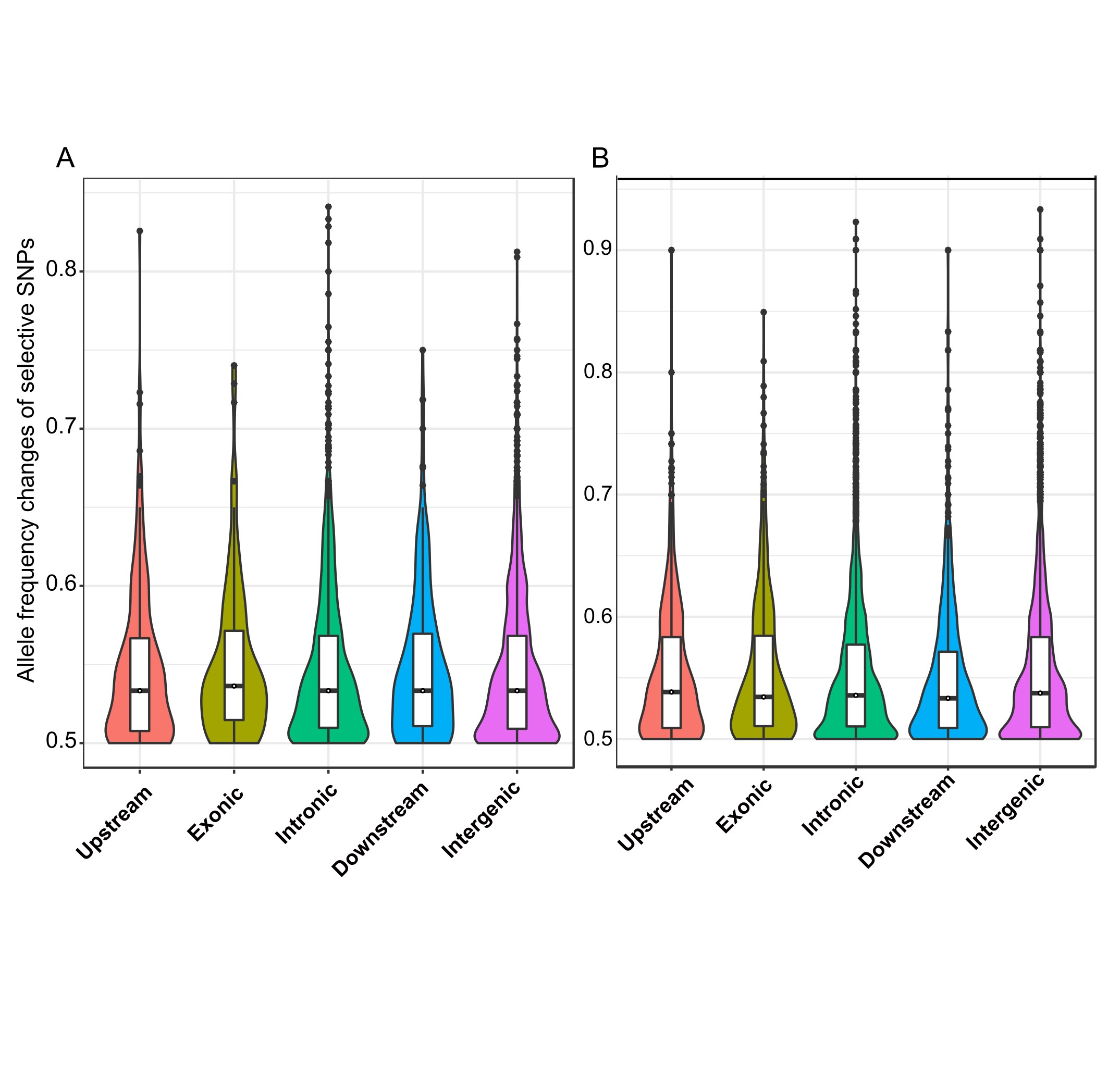

Supplement: Supplementary Figure 1 — Response of oyster larvae to OsHV-1 infection. Ratio of RNA-seq reads mapping to the OsHV-1 genome and the mortality of oyster larvae are shown (35) (the data refers to Huang et al,. 2017). [file DataSheet_1.zip › Supplementary Materials/Figure S3.jpg]

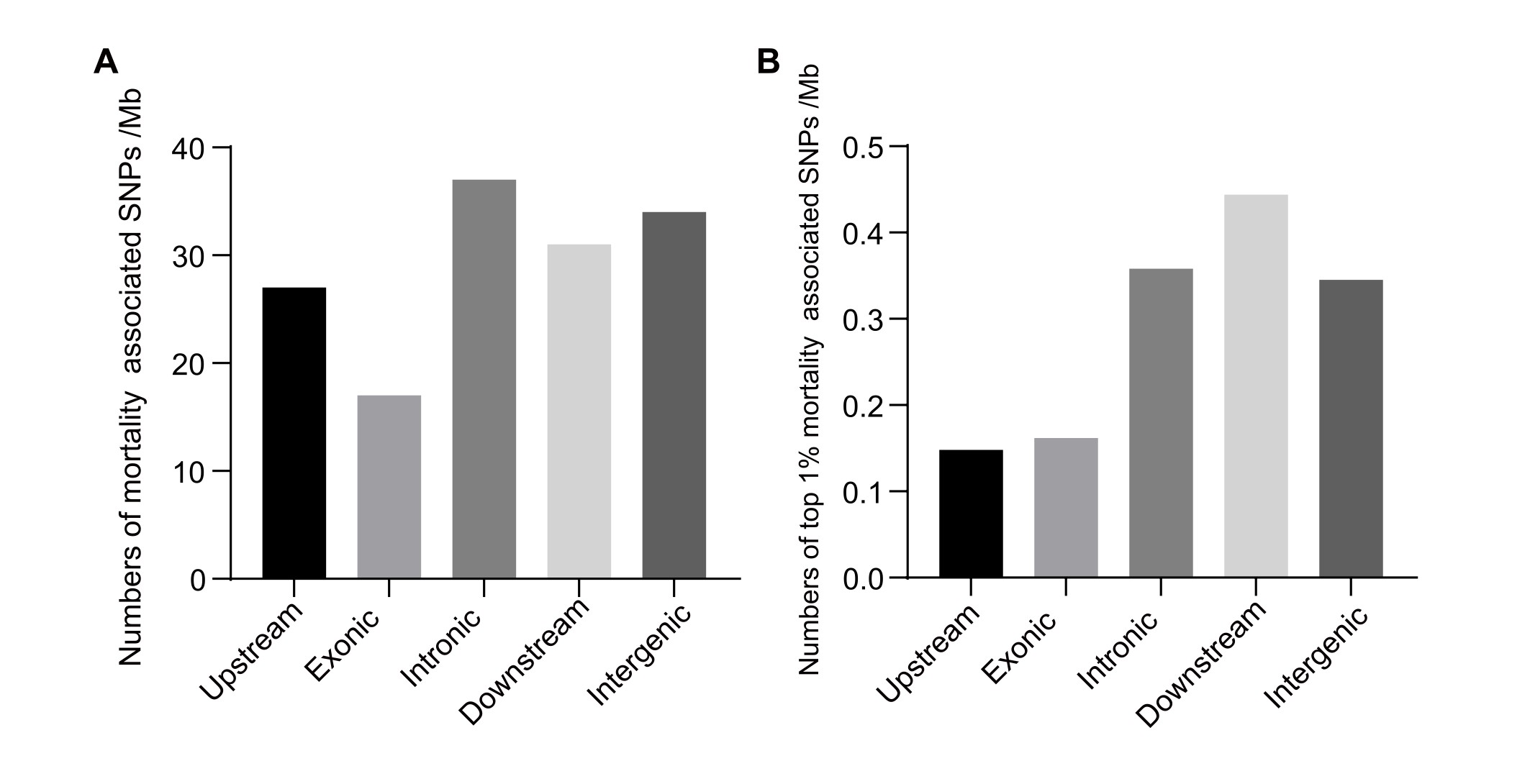

Supplement: Supplementary Figure 1 — Response of oyster larvae to OsHV-1 infection. Ratio of RNA-seq reads mapping to the OsHV-1 genome and the mortality of oyster larvae are shown (35) (the data refers to Huang et al,. 2017). [file DataSheet_1.zip › Supplementary Materials/Figure S4.jpg]
